# Prognostic significance of androgen receptor expression in breast cancer patients undergoing neoadjuvant chemotherapy: A retrospective cohort study

**DOI:** 10.1097/MD.0000000000050131

**Published:** 2026-08-14

**Authors:** Altay Aliyev, Adila Adilli, Aydan Ismayilsoy, Farida Aghayarli, Iqbal Babazade, Arturan Ibrahimli, Ceyhun Isayev, Thomas Karn, Bahar Gasimli, Khayal Gasimli

**Affiliations:** a Department of Oncology, Liv Bona Dea Hospital, Baku, Azerbaijan; b Department of Pathology, Liv Bona Dea Hospital, Baku, Azerbaijan; c Cleveland Clinic, Surgical Subspecialties Institute, Cleveland, OH; d Department of General Surgery, Liv Bona Dea Hospital, Baku, Azerbaijan; e Department of Obstetrics and Gynecology, Goethe University Frankfurt, University Hospital, Frankfurt, Germany.

**Keywords:** androgen receptor, breast cancer, neoadjuvant chemotherapy, disease-free survival, prognostic biomarker

## Abstract

The androgen receptor (AR) is an emerging biomarker in breast cancer (BC), yet its prognostic relevance in patients undergoing neoadjuvant chemotherapy (NACT) remains inconclusive. While most studies focus on Western populations, data from the Caucasus are scarce. Given regional differences in tumor biology and treatment access, evaluating AR’s clinical significance in these populations is crucial. This study aimed to investigate the association between AR expression, pathological response, and disease-free survival (DFS) in stage II to III BC patients receiving NACT. A retrospective cohort study was conducted on 132 female BC patients treated with NACT at Bonadea Hospital. AR expression patterns were categorized as negative, weak, moderate, or strong. Statistical analyses included chi-square tests, Kaplan–Meier survival analysis, and univariate Cox regression to evaluate associations between AR expression and clinicopathological features, pathological complete response (pCR), and DFS. The median age at diagnosis was 47 years. Invasive ductal carcinoma accounted for 91.1% of cases, with 50.0% of tumors graded as II or III. pCR was achieved in 28.6% of patients. Moderate-to-strong AR expression was observed in 15% of cases and was associated with a significantly lower Ki-67 index (*P* = .047). Although dichotomized AR expression did not significantly predict DFS (hazard ratio [HR]: 0.35, 95% confidence interval [CI]: 0.08–1.47, *P* = .151), Cox regression using all 4 AR categories revealed a significant reduction in recurrence risk (HR = 0.384, 95% CI: 0.160–0.918, *P* = .031). In multivariable model adjusting for estrogen receptor, human epidermal growth factor receptor 2, and Ki-67 expression, moderate-to-strong AR expression remained the most influential prognostic factor, although the association did not reach statistical significance (adjusted HR: 0.31, 95% CI: 0.07–1.41, *P* = .129; concordance index = 0.83). Among patients with residual disease (non-pCR), moderate-to-strong AR expression was associated with a higher 2-year DFS rate (90.9% vs 46.4%, *P* = .089). Sensitivity analysis demonstrated no evidence of selection bias related to AR data availability, as 2-year DFS was comparable between patients with and without available AR assessment (76.1% vs 71.6%, log-rank *P* = .975). Moderate-to-strong AR expression may be associated with improved DFS in patients treated with NACT, especially in those with incomplete pathological response. These findings highlight the potential of AR as a prognostic biomarker and therapeutic target, warranting validation in prospective studies.

## 1. Introduction

Breast cancer (BC) remains the most frequently diagnosed malignancy and a leading cause of cancer-related mortality among women globally.^[1]^ Neoadjuvant chemotherapy (NACT) has become an integral component of the treatment paradigm for patients with locally advanced BC, facilitating tumor downstaging and enhancing the feasibility of breast-conserving surgery.^[2]^ Beyond its therapeutic benefits, NACT also provides prognostic information based on the extent of treatment response. Notably, patients who fail to achieve a pathological complete response (pCR) remain at increased risk of recurrence, despite comparable long-term survival to those receiving adjuvant chemotherapy. Pathological complete response is widely accepted as a surrogate marker for favorable clinical outcomes, including improved disease-free survival (DFS) and overall survival (OS).^[3,4]^ Histopathological response is commonly evaluated using the Miller–Payne grading system, which assesses tumor response based on reductions in cellularity following therapy.^[5]^ Known predictors of pCR include a high Ki-67 proliferation index, estrogen receptor (ER) negativity, and high histologic grade.^[6–8]^ In recent years, the androgen receptor (AR) has emerged as a biomarker of growing interest in BC, particularly within the context of precision oncology. AR is expressed in approximately 70% to 90% of ER-positive tumors and 20% to 40% of triple-negative breast cancers (TNBC), positioning it as a potentially universal biomarker across molecular subtypes.^[9,10]^ The biological functions of AR are complex and context-dependent, with roles in tumor progression, therapeutic resistance, and cellular differentiation. While some studies have associated AR positivity with improved survival outcomes, others suggest a role in mediating resistance to endocrine therapies such as tamoxifen and aromatase inhibitors.^[11–14]^ These conflicting findings highlight the necessity for more refined characterization of AR’s clinical utility, particularly in the neoadjuvant setting. The impact of AR signaling on chemotherapy sensitivity remains incompletely understood. Preclinical and early-phase clinical evidence indicates that AR activation may confer resistance to conventional chemotherapy, an effect that may be reversible through targeted AR inhibition using agents such as enzalutamide.^[15]^ AR biology in BC intersects with several broader themes in molecular oncology. Recent reviews highlight the ubiquitous role of AR in non-prostatic hormone-responsive tumors including breast, salivary gland, and hepatocellular carcinomas^[16]^; the emerging clinical utility of AR-directed therapies such as enzalutamide, bicalutamide and next-generation AR-degraders in molecular apocrine and AR-driven TNBC^[17]^; and advances in immunohistochemical scoring standardization, digital image analysis and gene-expression-based classifiers.^[18–20]^ Together, these developments reinforce the need for cohort-level evidence on how AR expression relates to short-term treatment response and long-term prognosis in defined patient populations. Despite these promising findings, AR testing has not yet been incorporated into standard BC management protocols, and robust clinical evidence supporting its prognostic significance remains limited. Most existing data on AR expression derive from studies conducted in Western populations, leading to a paucity of evidence from underrepresented regions such as Central Asia and the Caucasus. Ethnogenomic variability, disparities in treatment access, and diagnostic heterogeneity may influence AR expression patterns and their clinical implications, underscoring the need for region-specific validation. In particular, the prognostic significance of AR in patients with residual disease post-NACT – a subgroup with substantial unmet clinical needs – remains inadequately explored. Despite growing global interest in AR as a prognostic marker, data from Central Asia and the Caucasus are virtually nonexistent. Given potential ethnic, genetic, and healthcare-related differences, regionally focused investigations are essential to confirm the broader applicability of AR-related prognostic insights.

In this context, we conducted a retrospective cohort study to examine the prognostic relevance of pretreatment AR expression in stage II to III BC patients treated with NACT. To our knowledge, this is the 1st study to assess the association between AR status, pathological response, and DFS in this population. We hypothesize that elevated AR expression is independently associated with improved DFS, particularly among patients with residual disease following NACT. Our findings aim to provide region-specific insights into the prognostic value of AR and to support future efforts toward more personalized approaches in BC management.

## 2. Materials and methods

### 2.1. Study design and patient selection

**Figure 1.**
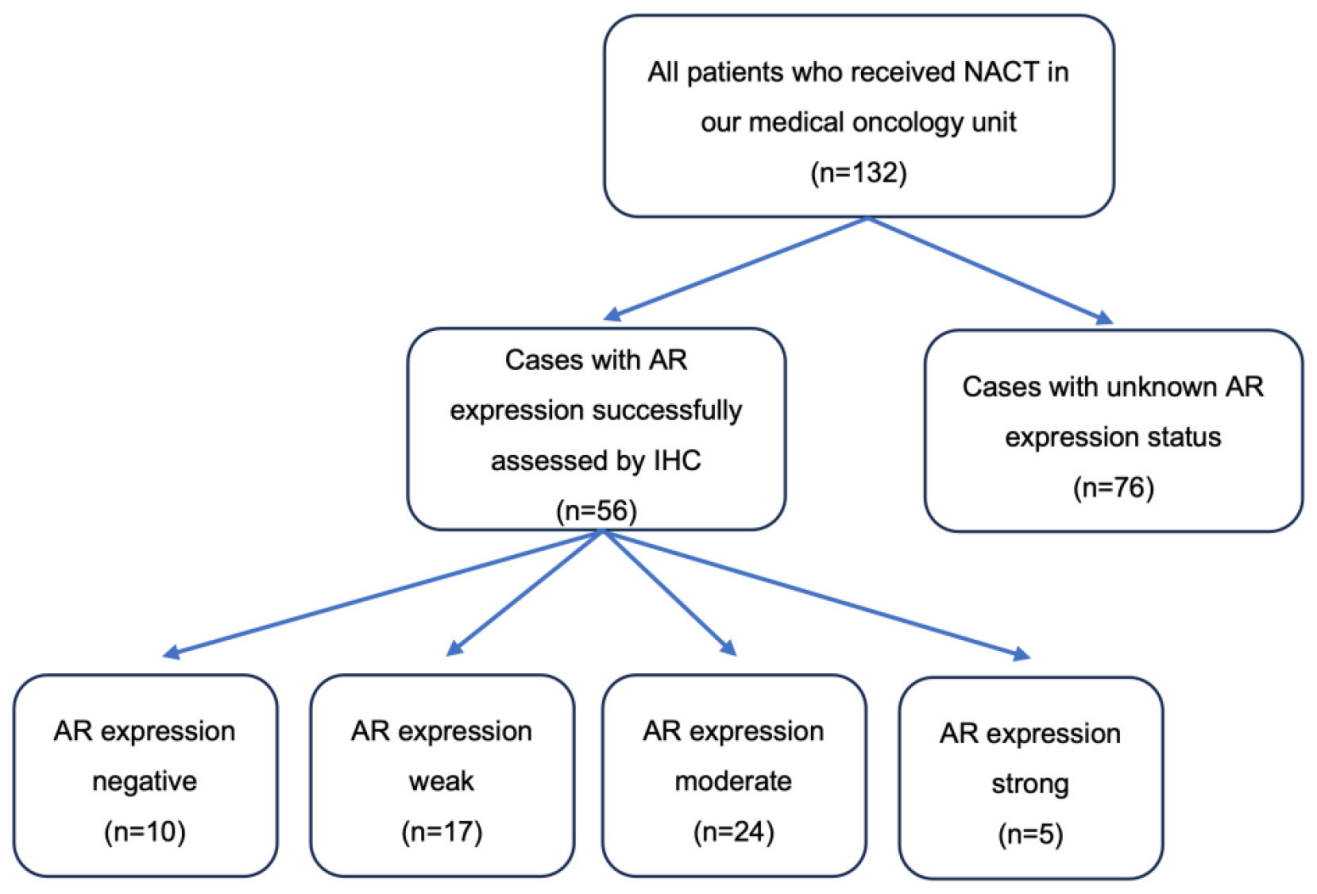
Flow chart of the study cohort. AR = androgen receptor, NACT = neoadjuvant chemotherapy.

This retrospective cohort study included 132 consecutive patients diagnosed with BC who underwent NACT in our hospital, between February 2020 and January 2024. Patient data were systematically retrieved from both digital and paper-based records maintained by the Department of Medical Oncology. All clinical and pathological data had been prospectively documented in the institutional cancer database. Patients with incomplete clinical or pathological data, missing histological subtype classification, or unconfirmed BC diagnosis were excluded. For the current analysis, we focused on 56 patients with available pretreatment AR status based on core needle biopsy (CNB) samples. The inclusion and exclusion process is detailed in the study flowchart (Fig. 1). To evaluate the potential for selection bias arising from the exclusion of 76 patients lacking pretreatment AR assessment, baseline clinicopathological characteristics and DFS were compared between patients with available AR data (n = 56) and those without (n = 76). No statistically significant differences were observed with respect to age, tumor grade, ER status, human epidermal growth factor receptor 2 (HER2) status, Ki-67 expression, or pCR rate. Likewise, 2-year DFS was nearly identical between the 2 groups (76.1% vs 71.6%; log-rank *P* = .975), arguing against clinically relevant selection bias.

### 2.2. Histopathological and clinical assessment

**Figure 2.**
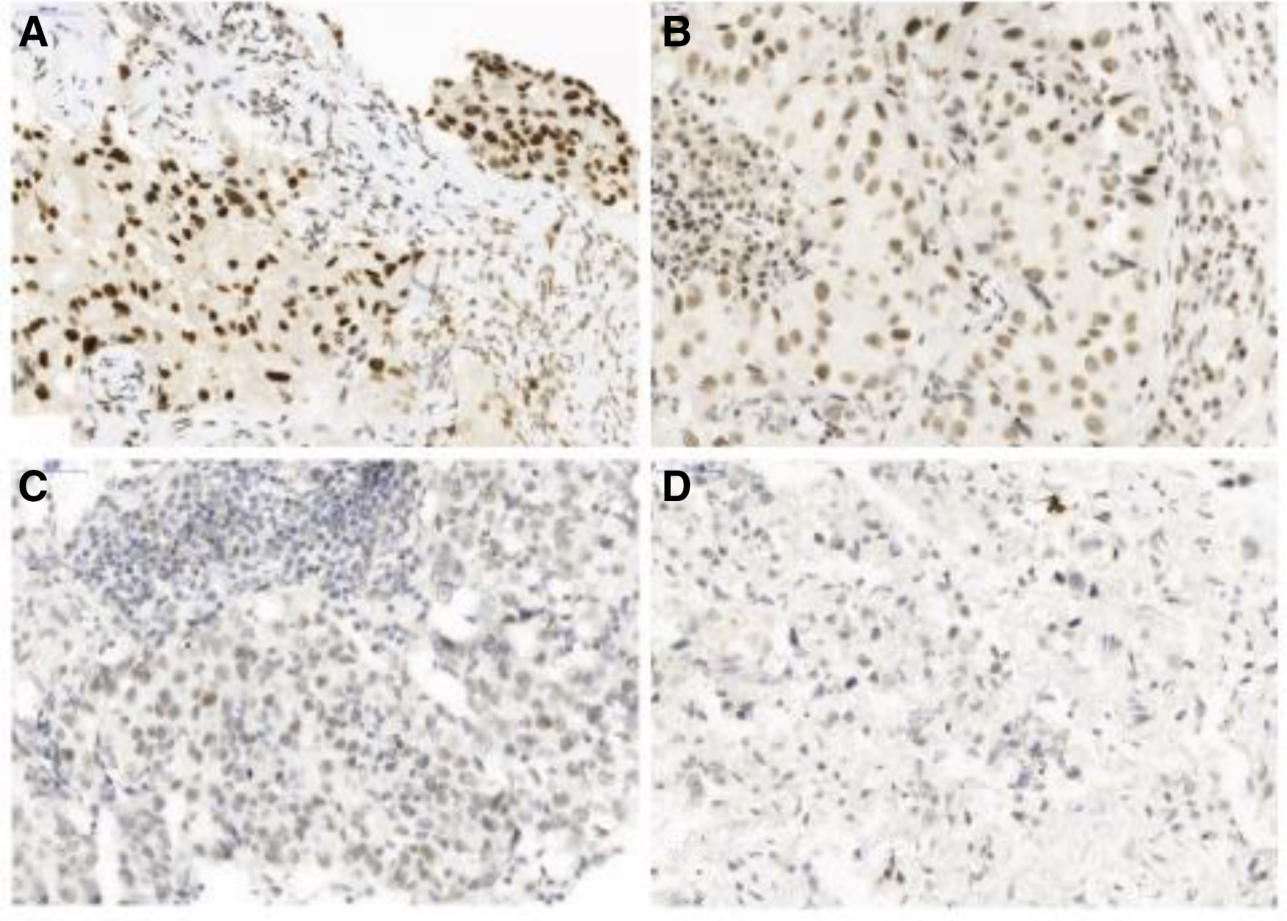
Representative immunohistochemical staining patterns of androgen receptor (AR) expression in invasive breast cancer tissue. (A) Strong nuclear staining (+++), (B) moderate nuclear staining (++), (C) weak nuclear staining (+), and (D) negative staining (0). AR expression was assessed on core needle biopsy specimens using IHC. IHC = immunohistochemical.

Histopathological evaluations and immunohistochemical (IHC) analyses were independently performed and reported by 2 board-certified pathologists at our institution. The diagnosis of malignancy was confirmed via CNB and resection specimens. Tumor staging (clinical T classification) was based on ultrasound examination of the breast and categorized according to the TNM classification system of the Union for International Cancer Control. For IHC assessment, tissue microarrays were constructed from archived formalin-fixed, paraffin-embedded tumor specimens. ER, partial response (PR), HER2, and AR status were assessed using IHC on a BenchMark Ultra Ventana platform (Ventana Medical Systems, a member of the Roche Group), following the manufacturer’s protocols. The primary antibodies used included: ER (clone SP1), PR (PatoLab, clone SP2), HER2 (C-erbB-2, clone e2-4001 + 3B5), and AR (clone AR441). ER and PR expression were interpreted according to the 2010 American Society of Clinical Oncology/College of American Pathologists guidelines, based on the percentage and intensity of nuclear staining. Samples were categorized as negative (<1% positive nuclei), low positive (1%–10%), or positive (>10%). HER2 status was assessed by IHC and scored from 0 to 3+ according to American Society of Clinical Oncology/College of American Pathologists criteria. A score of 0 or 1+ was defined as negative; 2+ as equivocal (requiring confirmation by in situ hybridization); and 3+ as positive. AR expression was semiquantitatively evaluated and classified into 4 categories based on nuclear staining intensity: negative (0), weak (+), moderate (++), and strong (+++) (Fig. 2). Each staining run included appropriate external positive and negative controls to ensure the quality and validity of the IHC analyses.

### 2.3. Patient stratification, pathological assessment, and data analysis

Patients were stratified based on AR expression levels assessed by IHC on pretreatment CNB specimens. Histopathological analyses were performed using standardized criteria to ensure diagnostic consistency. Parameters assessed included tumor subtype, histologic grade, and AR staining intensity. For statistical purposes, AR expression was dichotomized into low expression (negative or weak) and high expression (moderate or strong), to facilitate correlation with clinicopathological variables and survival outcomes. HER2 positivity was defined as IHC 3+ or IHC 2+ with in situ hybridization confirmation. pCR was defined as the absence of residual invasive carcinoma in both the breast and axillary lymph nodes (ypT0/is, ypN0). Comparative analyses were performed across AR expression subgroups for key clinicopathological variables, including age, body mass index, obesity status, comorbidities, histologic features, clinical stage, and type of systemic therapy. Survival analyses included assessments of DFS and OS, with a focus on prognostic factors associated with AR expression.

All treatment decisions regarding NACT were made following evaluation by a multidisciplinary BC board, in accordance with National Comprehensive Cancer Network guidelines. Following therapy initiation, patients entered a structured follow-up program that included periodic clinical and radiological assessments to evaluate treatment response and monitor for disease recurrence. Due to the retrospective nature of this study, the requirement for informed patient consent was waived.

### 2.4. Statistical analysis

All statistical analyses were conducted using IBM SPSS Statistics, version 29 (IBM Corp.). A 2-sided *P* value of <.05 was considered statistically significant. Survival curves were generated using the Kaplan–Meier method, and comparisons were made using the log-rank test. Cox proportional hazards models were employed to estimate hazard ratios (HRs) and 95% confidence intervals (CIs) for potential prognostic variables. Stepwise multivariate Cox regression analyses were performed to identify independent predictors of DFS and OS. In addition to the prespecified univariable analyses, an exploratory multivariable Cox proportional hazards model was fitted, including dichotomized AR expression, ER status, HER2 status, and Ki-67 expression as covariates selected a priori based on their established clinical relevance. Given the limited number of outcome events (n = 8), ridge penalization was applied to improve model stability, reduce coefficient inflation, and mitigate the risk of quasi-separation. To explore the consistency of the association between AR expression and clinical outcome across biologically distinct disease entities, prespecified subgroup analyses were performed according to molecular subtype (HR+/HER2−, HER2-positive, and TNBC). Categorical and continuous variables were compared using Pearson chi-square test or Fisher exact test, as appropriate.

## 3. Results

### 3.1. Baseline characteristics of the study cohort

The median age at diagnosis was 47 years (range: 25–76 years). Clinical tumor (cT) staging revealed that the majority of patients (82.1%) presented with cT2 tumors, while cT1, cT3, and cT4 tumors were observed in 7.1%, 5.4%, and 5.4% of cases, respectively. Regarding nodal involvement (clinical nodal [cN] stage), 44.7% of patients were diagnosed with cN2 disease, 23.2% with cN1 or cN3, and 8.9% with cN0. Histologically, invasive ductal carcinoma was the predominant subtype, accounting for 91.1% of cases, followed by invasive lobular carcinoma (7.1%) and papillary carcinoma (1.8%). Tumor grading was equally distributed between grade II (50.0%) and grade III (50.0%), with no grade I tumors observed. The Ki-67 index demonstrated a median value of 40% (range: 8%–90%). Hormone receptor status was determined on pretreatment CNB samples. ER expression was negative in 48.2% of cases, low (1%–10%) in 5.4%, and high (>10%) in 46.4%. PR was negative in 57.1%, low in 3.6%, and high in 39.3% of patients. HER2 status was negative in 69.6% and positive in 30.4% of patients. Clinical response to NACT included CR in 5.4%, PR in 87.4%, stable disease in 5.4%, and progressive disease in 1.8% of patients. pCR was achieved in 28.6%, while 71.4% exhibited residual disease (non-pCR) after NACT. In the non-pCR group, postoperative AR expression was negative in 30.0%, weak in 30.0%, moderate in 12.5%, and strong in 2.5% of patients. In 25.0% of non-pCR cases, AR evaluation was not feasible due to insufficient tissue. A comprehensive summary of baseline and histopathological characteristics is provided in Table 1.

**Table 1:** Baseline characteristics of the entire study cohort.

Variables	All patients, n = 56 (%)
Age at diagnosis (yr; median [range])	47 (25–76)
Menopause status	
Premenopausal	25 (44.6%)
Perimenopausal	9 (16.1%)
Postmenopausal	22 (39.3%)
Tumor localization	
Right	30 (53.6%)
Left	25 (44.6%)
Bilateral	1 (1.8%)
Tumor lesion	
Single	38 (67.9%)
Multifocal	6 (10.7%)
Multicentric	12 (21.4%)
Histologic subtype	
IDC	51 (91.1%)
ILC	4 (7.1%)
Papillary	1 (1.8%)
Grade	
I	0 (0%)
II	28 (50.0%)
III	28 (50.0%)
Ki-67 score (%; median [range])	40 (8–90)
Ki-67 score	
1–14	3 (5.4%)
15–30	22 (39.3%)
>30	31 (55.3%)
cT stage	
T1	4 (7.1%)
T2	46 (82.1%)
T3	3 (5.4%)
T4	3 (5.4%)
cN stage	
N0	5 (8.9%)
N1	13 (23.2%)
N2	25 (44.7%)
N3	13 (23.2%)
LVI status	
Negative	41 (73.2%)
Positive	15 (26.8%)
ER expression (%, pretherapeutic)	
0	27 (48.2%)
1–10	3 (5.4%)
>10	26 (46.4%)
PR expression (%, pretherapeutic)	
0	32 (57.1%)
1–10	2 (3.6%)
>10	22 (39.3%)
HER2 status (pretherapeutic)	
Negative	39 (69.6%)
Positive*	17 (30.4%)
Response to NACT	
CR	3 (5.4%)
PR	49 (87.4%)
SD	3 (5.4%)
PD	1 (1.8%)
pCR status	
No	40 (71.4%)
Yes	16 (28.6%)
RCB	
0	19 (33.9%)
1	3 (5.4%)
2	15 (26.8%)
3	19 (33.9%)
AR expression (postoperative, in non-pCR)	
Negative	12 (30.0%)
Weak	12 (30.0%)
Moderate	5 (12.5%)
Strong	1 (2.5%)
n.a.†	10 (25.0%)

AR = androgen receptor, cN = clinical nodal, CR = complete response, cT = clinical tumor, ER = estrogen receptor, HER2 = human epidermal growth factor receptor 2, IDC = invasive ductal carcinoma, ILC = invasive lobular carcinoma, LVI = lymphovascular invasion, n.a = not applicable, NACT = neoadjuvant chemotherapy, pCR = pathological complete response, PR = progesterone receptor, RCB = residual cancer burden, SD = stable disease.

* HER2 positivity definded as HER2 overexpression in immunohistochemical staning of 3+ or positivity of fluorescence in situ hybridization in IHC 2+ cases.

† The IHC staining could not be performed due to the insufficient amount of material.

### 3.2. Comparison of clinicopathological features by AR expression

The median age was slightly lower in patients with moderate/strong AR expression (40 years; range: 25–76) compared to those with negative/weak expression (48 years; range: 32–72), though this difference was not statistically significant (*P* = .250). A trend toward a higher prevalence of invasive lobular carcinoma was noted in the moderate/strong AR group (13.8%) versus 0% in the negative/weak group (*P* = .078). The Ki-67 index was numerically lower in the moderate/strong AR group (median: 30%, range: 10–90) compared to the negative/weak group (median: 45%, range: 8–85). Although this difference reached nominal statistical significance in the univariable Mann–Whitney *U* test (*P* = .047), the small sample size (n = 56) and the borderline *P* value warrant cautious interpretation, and the finding should be considered exploratory and hypothesis-generating rather than confirmatory. Importantly, in the multivariable Cox proportional hazards model presented below, Ki-67 was not independently associated with DFS (adjusted HR per 1% increase, 1.00; 95% CI: 0.96–1.03; *P* = .813), indicating that the apparent univariable association was not robust after adjustment for established clinicopathological covariates. Lymphovascular invasion was more frequent in the moderate/strong AR group (34.5% vs 18.5%), although this trend did not reach statistical significance (*P* = .178). HER2 positivity was more common in the moderate/strong AR group (37.9%, 11/29) than in the negative/weak group (22.2%, 6/27), although this difference did not reach statistical significance (*P* = .324). Among non-pCR patients, AR negativity was more prevalent in the negative/weak group (50.0%) than in the moderate/strong group (28.6%) (*P* = .492). Detailed statistical comparisons are presented in Table 2.

**Table 2:** Clinicopathological features stratified by AR expression pattern.

Variables	AR negative/weak, n = 27 (%)	AR moderate/strong, n = 29 (%)	*P* value
Age at diagnosis (yr; median [range])	48 (32–72)	40 (25–76)	.250
Menopause status			.381
Premenopausal	10 (37.0%)	15 (51.7%)	
Perimenopausal	6 (22.2%)	3 (10.3%)	
Postmenopausal	11 (40.7%)	11 (37.9%)	
Tumor localization			.402
Right	13 (48.1%)	17 (58.6%)	
Left	14 (51.9%)	11 (37.9%)	
Bilateral	0 (0%)	1 (3.4%)	
Tumor lesion			.601
Single	17 (63.0%)	21 (72.4%)	
Multifocal	4 (14.8%)	2 (6.9%)	
Multicentric	6 (22.2%)	6 (20.7%)	
Histologic subtype			.078
IDC	27 (100%)	24 (82.8%)	
ILC	0 (0%)	4 (13.8%)	
Papillary	0 (0%)	1 (3.4%)	
Grade			.789
I	0 (0%)	0 (0%)	
II	13 (48.1%)	15 (51.7%)	
III	14 (51.9%)	14 (48.3%)	
Ki-67 score (%; median [range])	45 (8–85)	30 (10–90)	**.047**
Ki-67 score			.137
1–14	2 (7.4%)	1 (3.4%)	
15–30	7 (25.9%)	15 (51.7%)	
>30	18 (66.7%)	13 (44.8%)	
cT stage			
T1	2 (7.4%)	2 (6.9%)	.897
T2	23 (85.2%)	23 (79.3%)	
T3	1 (3.7%)	2 (6.9%)	
T4	1 (3.7%)	2 (6.9%)	
cN stage			
N0	2 (7.4%)	3 (10.3%)	.739
N1	6 (22.2%)	7 (24.1%)	
N2	14 (51.9%)	11 (37.9%)	
N3	5 (18.5%)	8 (27.6%)	
LVI status			.178
Negative	22 (81.5%)	19 (65.5%)	
Positive	5 (18.5%)	10 (34.5%)	
ER expression (%, pretherapeutic)			.545
0	15 (55.6%)	12 (41.4%)	
1–10	1 (3.7%)	2 (6.9%)	
>10	11 (40.7%)	15 (51.7%)	
PR expression (%, pretherapeutic)			.946
0	16 (59.3%)	16 (55.2%)	
1–10	1 (3.7%)	1 (3.4%)	
>10	10 (37.0%)	12 (41.4%)	
HER2 status (pretherapeutic)			.324
Negative	21 (77.8%)	18 (62.1%)	
Positive*	6 (22.2%)	11 (37.9%)	
Response to NACT			.232
CR	0 (0%)	3 (10.3%)	
PR	25 (92.6%)	24 (82.8%)	
SD	2 (7.4%)	1 (3.4%)	
PD	0 (0%)	1 (3.4%)	
pCR status			.866
No	19 (70.4%)	21 (72.4%)	
Yes	8 (29.6%)	8 (27.6%)	
RCB			.930
0	8 (29.6%)	11 (37.9%)	
1	1 (3.7%)	2 (6.9%)	
2	7 (25.9%)	8 (27.5%)	
3	11 (40.7%)	8 (27.5%)	
AR expression (postoperative, in non-pCR)			.492
Negative	8 (50.0%)	4 (28.6%)	
Weak	6 (37.5%)	6 (42.9%)	
Moderate	2 (12.5%)	3 (21.4%)	
Strong	0 (0%)	1 (7.1%)	

Bold indicates statistically significance.

AR = androgen receptor, cN = clinical nodal, CR = complete response, cT = clinical tumor, ER = estrogen receptor, HER2 = human epidermal growth factor receptor 2, IDC = invasive ductal carcinoma, ILC = invasive lobular carcinoma, LVI = lymphovascular invasion, n.a = not applicable, NACT = neoadjuvant chemotherapy, pCR = pathological complete response, PR = progesterone receptor, RCB = residual cancer burden, SD = stable disease.

* HER2 positivity definded as HER2 overexpression in immunohistochemical staning of 3+ or positivity of fluorescence in situ hybridization in IHC 2+ cases.

### 3.3. Survival analysis

The median follow-up period was 12 months (range: 5–40 months). During this time, 8 patients experienced disease recurrence and 2 AR-evaluable patients died from disease progression. The median DFS was not reached for the entire cohort, with an estimated 2-year DFS rate of 76.1% (±8.7%). In univariate Cox regression analysis, AR expression, when analyzed as a 4-tier variable (negative, weak, moderate, and strong), was significantly associated with recurrence risk (HR = 0.384; 95% CI: 0.160–0.918; *P* = .031). However, dichotomizing AR expression into low (negative/weak) versus high (moderate/strong) categories did not yield a statistically significant association with DFS (HR: 0.35, 95% CI: 0.08–1.47, *P* = .151). Other clinicopathological parameters – including ER status (*P* = .181), menopausal status (*P* = .761), body mass index (*P* = .981), HER2 status (*P* = .270), tumor grade (*P* = .894), pCR status (*P* = .327), and Ki-67 index (*P* = .266) - were not significantly associated with DFS. The results of the univariate analysis are detailed in Table 3.

**Table 3:** Univariate Cox regression analysis of disease-free survival based on androgen receptor expression in pretreatment biopsies.

Variable	HR	95% CI	*P* value
Menopause status	0.888	0.412–1.911	.761
BMI	0.991	0.455–2.156	.981
AR per grade (negative, low, moderate, and strong)	0.384	0.160–0.918	**.031**
AR* (moderate/strong vs negative/low)	0.346	0.081–1.473	.151
ER (negative vs positive)	0.334	0.067–1.663	.181
HER2† (positive vs negative)	0.618	0.263–1.452	.270
pCR (vs. non-pCR)	0.350	0.043–2.850	.327
Grading	1.099	0.272–4.432	.894
Ki-67 score (low, medium, and high)	2.372	0.518–10.872	.266
HER2 status	0.618	0.263–1.452	.270

Bold indicates statistically significance.

AR = androgen receptor, BMI = body mass index, CI = confidence interval, ER = estrogen receptor, HER2 = human epidermal growth factor receptor 2, HR = hazard ratio, pCR = pathological complete response.

* A dichotomic classification of AR expression.

† HER2 positivity definded as HER2 overexpression in immunohistochemical staning of 3+ or positivity of fluorescence in situ hybridization in IHC 2+ cases.

**Figure 3.**
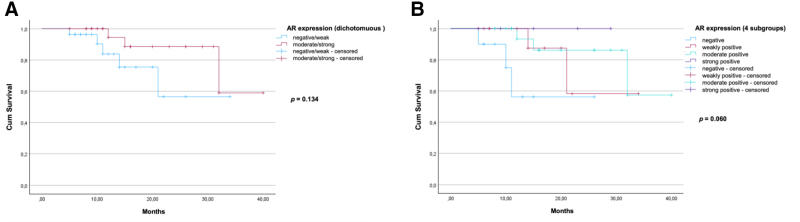
Kaplan–Meier curves for disease-free survival assessing the prognostic value of the androgen receptor based on its expression pattern, classified both dichotomously (A) and as individual expression levels (B). AR = androgen receptor.

**Figure 4.**
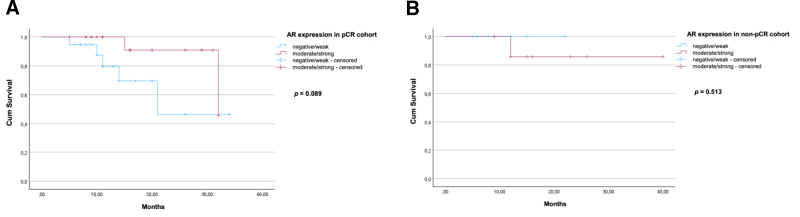
Subgroup analysis of disease-free survival assessing the prognostic value of androgen receptor status in patients with non-pCR (A) and pCR (B). AR = androgen receptor, pCR = pathological complete response.

Kaplan–Meier survival analysis (Fig. 3) demonstrated that, using a dichotomous classification, the 2-year DFS was 56.6% (±18.4%) in the low AR group and 88.5% (±7.6%) in the high AR group (*P* = .134). When stratified by 4 AR categories, 2-year DFS rates were: AR-negative, 56.3% (±20.1); weak, 58.3% (±25.1); moderate, 86.2% (±9.1); and strong, with no observed events (*P* = .060). Subgroup analysis by pCR status revealed generally favorable outcomes in patients who achieved pCR, with only 1 recurrence. DFS did not differ significantly between AR subgroups in this cohort (*P* = .513; Fig. 4B). Conversely, in the non-pCR group, the 2-year DFS was significantly higher in patients with moderate/strong AR expression (90.9% ±8.7) compared to those with negative/weak expression (46.4% ±20.9) (*P* = .089; Fig. 4A), indicating a potential prognostic relevance of AR in the context of residual disease after NACT.

**Figure 5.**
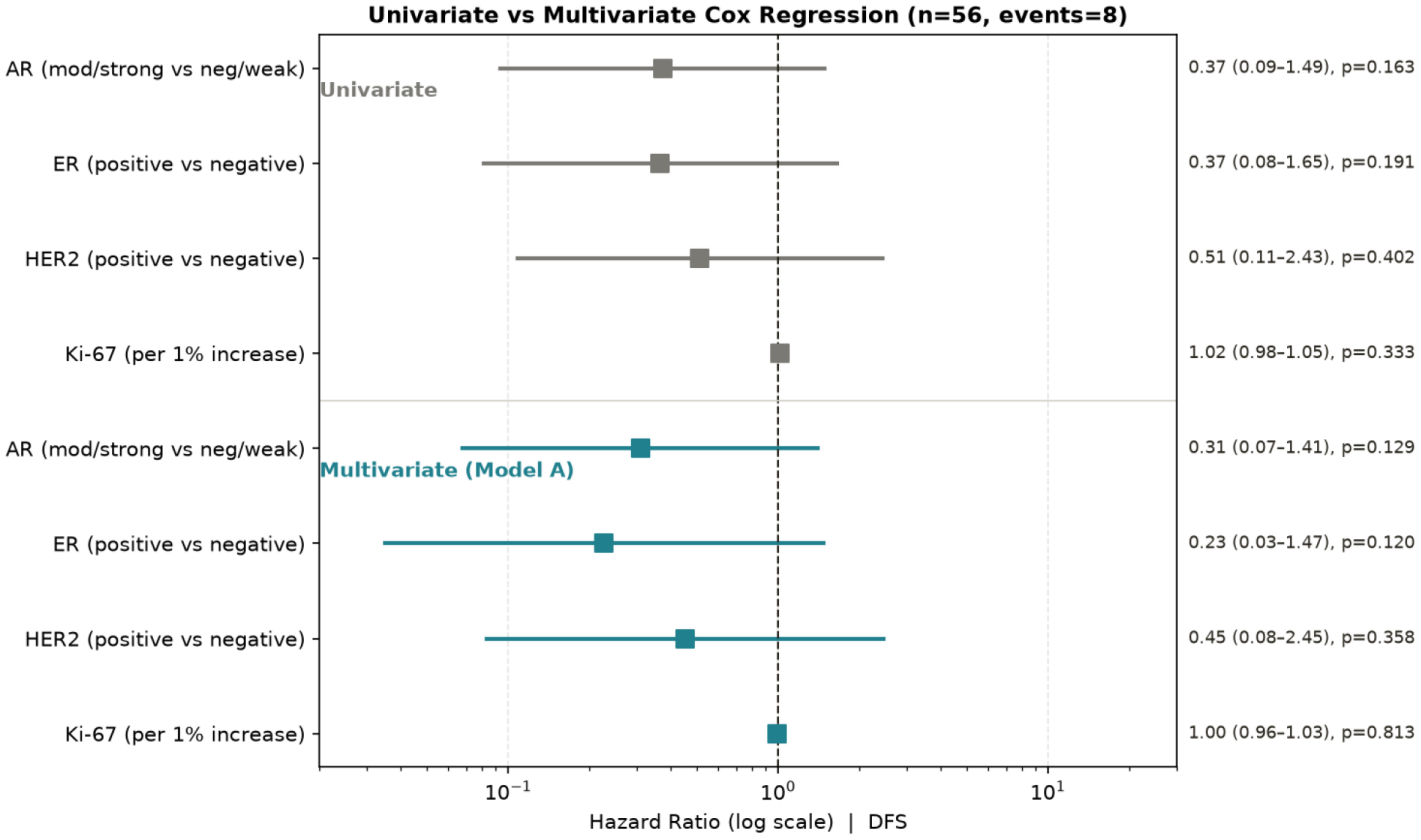
Univariate versus multivariate Cox regression analysis of disease-free survival including different biomarkers. AR = androgen receptor, DFS = disease-free survival, ER = estrogen receptor, HER2 = human epidermal growth factor receptor 2.

To investigate whether the prognostic association of AR expression is independent of established clinicopathological biomarkers, an exploratory multivariable Cox proportional hazards model was fitted including dichotomized AR expression, ER status (≥10% vs <10%), HER2 status, and Ki-67 expression (continuous) as covariates. As illustrated in Figure 5, moderate-to-strong AR expression remained the strongest prognostic factor for DFS, although the association did not reach statistical significance (adjusted HR: 0.31; 95% CI: 0.07–1.41; *P* = .129). Likewise, ER status (adjusted HR: 0.23; 95% CI: 0.03–1.47; *P* = .120), HER2 status (adjusted HR: 0.45; 95% CI: 0.08–2.45; *P* = .358), and Ki-67 expression (per 1% increase: adjusted HR: 1.00; 95% CI: 0.96–1.03; *P* = .813) were not independently associated with DFS. The model demonstrated good discriminative performance, with a concordance index of 0.83, indicating a high ability of the combined biomarker model to distinguish patients according to their risk of recurrence. Given the limited number of outcome events (n = 8), however, the estimated hazard ratios should primarily be interpreted as measures of effect size rather than definitive evidence of independent prognostic significance.

In prespecified subgroup analyses stratified by molecular subtype (HR+/HER2−, n = 18; HER2-positive, n = 17; TNBC, n = 21), moderate-to-strong AR expression was consistently associated with numerically higher 2-year DFS rates across all subtypes: 100% versus 80% (*P* = .937) in HR+/HER2− disease, 88.9% versus 50.0% (*P* = .327) in HER2-positive disease, and 80.0% versus 62.9% (*P* = .249) in TNBC. Although none of these subgroup comparisons reached statistical significance because of the very limited number of events (2–4 per subgroup), the consistent direction of the observed effect across biologically distinct BC subtypes supports the hypothesis that the prognostic relevance of AR expression is unlikely to be solely attributable to its association with a particular molecular subtype.

## 4. Discussion

Androgens are physiologically present in breast tissue and have been detected at elevated levels in both ductal carcinoma in situ and invasive breast carcinoma. In recent years, the AR has garnered increasing clinical interest, primarily due to its emerging potential as a therapeutic target. AR expression has been reported in approximately 60% to 90% of BC cases, with the highest prevalence observed in ER-positive subtypes (~70%) and in around 40% of TNBC.^[21–23]^ In this retrospective cohort study involving 132 BC patients treated with NACT, we conducted a comprehensive evaluation of various clinicopathological parameters to determine their prognostic significance, with a particular focus on AR expression. Among the factors analyzed, AR expression emerged as a significant independent predictor of DFS, especially in patients who did not achieve a pCR. Previous studies have suggested that AR expression may be associated with a more favorable prognosis in certain BC subtypes, particularly ER-positive tumors^[24]^ and molecular apocrine-like phenotypes.^[25]^ However, our cohort represented a biologically heterogeneous population, including a substantial proportion of ER-negative and HER2-positive tumors. Despite this variability, we observed a consistent trend toward improved DFS among patients with moderate-to-strong AR expression, most notably within the subgroup that did not achieve pCR. In this subgroup, the 2-year DFS rate was 90.9% in patients with high AR expression compared to 46.4% in those with low AR expression, suggesting a potential protective role of AR, even in the absence of pCR. This observation is further supported by a large meta-analysis by Bozovic-Spasojevic et al, which included over 17,000 early-stage BC patients and demonstrated a significant association between AR positivity and improved OS and DFS, independent of ER status.^[22]^

Consistent with these findings, our study demonstrated a significant association between AR expression and improved DFS in univariable Cox regression analysis (HR = 0.38; 95% CI: 0.16–0.91; *P* = .031). Although Kaplan–Meier survival analysis showed a similar trend, the difference narrowly missed statistical significance (*P* = .060).

In our cohort, tumors with moderate-to-strong AR expression exhibited a numerically lower Ki-67 index than those with negative or weak AR expression (*P* = .047 in univariate testing). This observation is consistent with previous reports suggesting an inverse relationship between AR expression and tumor proliferative activity. Notably, the meta-analysis by Vera-Badillo et al, comprising 7693 patients from 19 studies, demonstrated a strong association between AR positivity and lower Ki-67 expression (chi-square *P* < .001).^[26]^ However, the magnitude and consistency of this association have varied substantially across studies. The larger Nurses’ Health Study cohort reported by Kensler et al (n = 354) identified only a weak inverse correlation between AR expression and Ki-67 (Spearman rho = −0.09),^[27]^ whereas several other IHC studies failed to detect a significant association altogether.^[28,29]^ These discrepancies likely reflect differences in patient populations, molecular subtype distribution, AR and Ki-67 assessment methodologies, and positivity thresholds across studies. In the present study, the borderline statistical significance and absence of an independent association between Ki-67 and DFS in the multivariable Cox model (adjusted HR: 1.00 per 1%, *P* = .813) argue against overinterpretation of this finding. Accordingly, the observed inverse association between AR expression and Ki-67 should be regarded as exploratory and hypothesis-generating rather than as evidence of a definitive biological relationship.

If confirmed in larger, adequately powered cohorts, reduced proliferative activity in AR-positive tumors could represent 1 biological mechanism underlying the consistently lower pCR rates reported in AR-positive hormone receptor–positive and TNBC.^[23]^ However, the present study was not sufficiently powered to address this mechanistic hypothesis.

In TNBC, AR positivity defines a distinct molecular apocrine-like subtype characterized by AR-driven signaling in the absence of ER expression. This biologically unique subtype exhibits distinct transcriptional profiles that may underlie its relative resistance to chemotherapy. Supporting this notion, Shi et al reported significantly lower pCR rates following NACT in AR-positive TNBC patients.^[27]^ Similarly, although AR-positive TNBC cases have been associated with significantly lower pCR rates compared to AR-negative cases (12.8% vs 25.4%),^[28]^ accumulating evidence suggests that AR expression may be linked to improved long-term outcomes, including DFS and OS.^[29–31]^ Our findings align with this apparent paradox: despite lower pCR rates, patients with moderate-to-strong AR expression showed a markedly higher 2-year DFS, particularly within the non-pCR subgroup. This supports the hypothesis that AR may exert a protective effect independent of immediate treatment response.

In HR-positive breast tumors, AR expression has been associated with reduced cellular proliferation and potentially decreased chemosensitivity. This observation is consistent with our results, wherein AR-positive tumors exhibited significantly lower Ki-67 indices, indicating a less aggressive phenotype that may nonetheless be less responsive to cytotoxic agents. Similar findings were reported by Cochrane et al, who demonstrated that AR activation modulates transcriptional programs that promote cell survival while suppressing proliferation.^[15]^ The observed antiproliferative effect of AR may be partly attributed to the complex interplay between androgen and ER signaling pathways. AR has been shown to compete with ER for binding to estrogen response elements, thereby attenuating ER-mediated transcription and limiting estrogen-driven proliferation.^[32]^ Moreover, the enzymatic conversion of androgens to estrogens adds an additional layer of complexity to this hormonal axis. Notably, tumors co-expressing both ER and AR are often associated with more favorable clinical outcomes than those expressing ER alone, possibly due to AR-mediated antagonism of ER signaling.^[15,33]^ These findings support the notion of AR as a context-dependent modulator, whose prognostic impact is shaped by the tumor’s hormonal receptor profile.

The prognostic role of AR in HER2-positive BC remains controversial. While studies such as that by Akashi et al reported higher pCR rates in AR-positive HER2+ tumors, other evidence points to an association between AR expression and poorer outcomes.^[34]^ This discrepancy is further highlighted by a pooled analysis by Venema et al, which found that elevated AR mRNA expression in HER2+ tumors correlated with inferior DFS.^[35]^ In our cohort, no statistically significant associations were observed between AR expression and key clinicopathological parameters such as tumor grade, HER2 status, or ER expression.

The numerically lower Ki-67 index in AR-positive tumors (*P* = .047) is consistent with previous reports linking AR expression to greater differentiation and lower proliferative activity,^[29]^ but should be interpreted as exploratory given the borderline statistical significance and limited sample size.

Although univariate Cox regression using a 4-tier classification of AR expression showed a statistically significant association with DFS, dichotomization into high versus low AR expression did not reach significance. This may be due to limited statistical power, especially in the high AR subgroup, where no DFS events occurred. Nevertheless, Kaplan–Meier analysis showed a clear trend toward improved DFS with increasing AR expression, reinforcing its potential clinical relevance.

Subgroup analyses provided additional insights, particularly among non-pCR patients, where AR expression appeared to stratify prognosis more distinctly. Since patients who fail to achieve pCR after NACT are generally at higher risk of recurrence, the ability of AR expression to identify a subset with more favorable outcomes is of clinical interest. Biomarkers such as AR may aid in refining prognostic assessments and guiding individualized post-NACT therapeutic strategies.

Overall, our findings highlight the complex, subtype-specific prognostic implications of AR expression in BC. The associations between AR expression, lower Ki-67 indices, and improved DFS suggest that AR may serve as a valuable prognostic biomarker, particularly in patients with residual disease following NACT. These insights also provide a rationale for further exploration of AR-targeted therapeutic approaches in appropriately selected patient subgroups.

This study has several limitations. First, its retrospective design may introduce selection bias. This study has several limitations. First, its retrospective design may have introduced selection bias. To address this, a sensitivity analysis demonstrated no significant differences in baseline characteristics or DFS between patients with and without available AR data (log-rank *P* = .975), arguing against systematic selection. Second, the limited sample size restricted statistical power, particularly for multivariable and subgroup analyses, which should therefore be considered exploratory. Third, the cohort was biologically heterogeneous. Although subgroup analyses stratified by HR/HER2 subtype showed consistent effect directions, the small number of events precluded definitive subtype-specific conclusions, underscoring the need for validation in larger, subtype-specific cohorts. Finally, the median follow-up of 12 months is relatively short for a DFS endpoint, and longer follow-up is required to confirm the observed associations. Despite its limitations, this study constitutes the 1st comprehensive evaluation of AR expression in BC patients receiving NACT in the Caucasus region, thereby contributing important data from a previously underrepresented population. Future studies with larger, subtype-specific cohorts are warranted to validate these findings and to further elucidate the molecular mechanisms linking AR signaling to BC progression.

In conclusion, our findings suggest that pretherapeutic AR expression may serve as a prognostic marker in BC patients undergoing NACT, particularly among those who do not achieve pCR. Incorporating AR assessment into routine pretreatment diagnostics may improve prognostic precision and support the development of risk-adapted, personalized therapeutic strategies.

## Acknowledgments

We extend our sincere appreciation to the patients, their families, and caregivers for their invaluable participation in this study. We also wish to acknowledge the investigators and site personnel for their commitment and essential contributions. Furthermore, we are grateful to the Mildred-Scheel-Nachwuchszentrum (MSNZ) Frankfurt for its support, particularly through the Excellence Program for the Advancement of Scientific Research and Early-Career Scientists, which has played a pivotal role in supporting the work of Dr Khayal Gasimli.

## Author contributions

**Conceptualization:** Altay Aliyev.

**Data curation:** Aydan Ismayilsoy, Farida Aghayarli, Iqbal Babazade, Arturan Ibrahimli, Ceyhun Isayev.

**Formal analysis:** Adila Adilli, Ceyhun Isayev, Thomas Karn.

**Investigation:** Adila Adilli, Iqbal Babazade, Ceyhun Isayev.

**Methodology:** Adila Adilli.

**Software:** Thomas Karn.

**Supervision:** Khayal Gasimli.

**Validation:** Altay Aliyev.

**Writing – original draft:** Bahar Gasimli.

**Writing – review & editing:** Altay Aliyev, Arturan Ibrahimli.
